# Gate-controlled electromechanical backaction induced by a quantum dot

**DOI:** 10.1038/ncomms11132

**Published:** 2016-04-11

**Authors:** Yuma Okazaki, Imran Mahboob, Koji Onomitsu, Satoshi Sasaki, Hiroshi Yamaguchi

**Affiliations:** 1 NTT Basic Research Laboratories, NTT Corporation, 3-1 Morinosato-Wakamiya, Atsugi, Kanagawa 243-0198, Japan

## Abstract

Semiconductor-based quantum structures integrated into mechanical resonators have emerged as a unique platform for generating entanglement between macroscopic phononic and mesocopic electronic degrees of freedom. A key challenge to realizing this is the ability to create and control the coupling between two vastly dissimilar systems. Here, such coupling is demonstrated in a hybrid device composed of a gate-defined quantum dot integrated into a piezoelectricity-based mechanical resonator enabling milli-Kelvin phonon states to be detected via charge fluctuations in the quantum dot. Conversely, the single electron transport in the quantum dot can induce a backaction onto the mechanics where appropriate bias of the quantum dot can enable damping and even current-driven amplification of the mechanical motion. Such electron transport induced control of the mechanical resonator dynamics paves the way towards a new class of hybrid semiconductor devices including a current injected phonon laser and an on-demand single phonon emitter.

## Abstract (web summary)

Coupling between quantum structures and mechanical resonators remains a challenge. Here, the authors couple a quantum dot and a piezoelectric microresonator and show that gate-induced single electron transport in the quantum dot enables control of the amplitude of the mechanical response.

## Graphical abstract

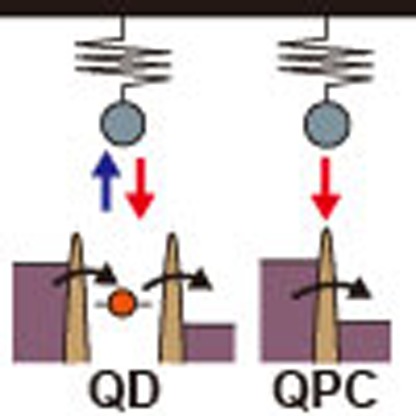

Mechanical resonators are one of the most ideal realization of harmonic oscillators with excellent quality factors of >10^6^ and high operation frequencies that can be >1 GHz, and they have emerged as an important platform for both scientific studies and new device applications^1^. The key to further functionalizing the resonator is its hybridization with other physical systems, where the resonator motion can be not only detected but also manipulated by the auxiliary system using backaction from the coupling. One of the most successful platforms for this concept are cavity optomechanics where the mechanical resonator forms one of the mirrors in an optical cavity and the resultant backaction force from the confined photons can damp and amplify its harmonic motion^2^,^3^. This reversible feature plays an essential role in many applications, especially in the generation of non-classical phonon states^4^,^5^, where cooling and the subsequent parametric amplification allow preparation of entangled states superposing both phonons and photons^6^,^7^.

Compared with optomechanical systems, the hybridization of a mechanical resonator with a quantum low-dimensional system has been barely developed despite its importance for many electromechanical applications^8^,^9^,^10^,^11^,^12^,^13^,^14^,^15^,^16^,^17^. This is principally due to the integration of an electron cavity, that is, a quantum dot (QD), into the resonator with perfectly controlled coupling proving technologically challenging. Previously, such hybrid devices have been studied using primarily metal-based single electron transistors (SETs), in which the tunnel barriers and the electron energies lack the wide tunability required for creating and harnessing the precisely controlled electron backaction onto the mechanics^18^,^19^,^20^,^21^,^22^,^23^,^24^.

In this study, we have employed a gate-controlled QD based on the GaAs/AlGaAs system, which enables superior control of the confined electron states, and the intrinsic piezoelectricity of this material system allows highly efficient electron-to-mechanics coupling. With the aid of this QD-resonator platform, precise control of the backaction polarity and magnitude is demonstrated by only adjusting the operation point of the QD gate bias.

## Results

### Hybrid mechanical resonator QD system

**Figure 1:**
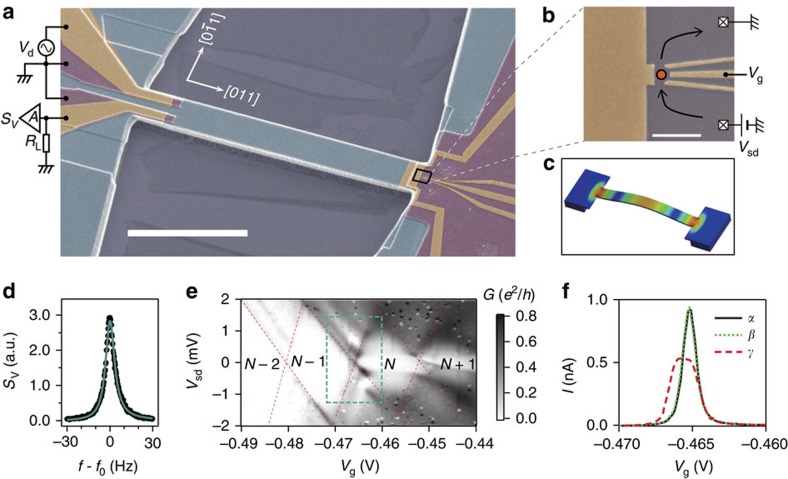
Hybrid mechanical resonator quantum dot system. (**a**) False-colour scanning electron microscope (FCSEM) image of the hybrid device along with the measurement setup (scale bar, 20 μm). A doubly clamped electromechanical resonator of 50 μm length, 6 μm width and 1 μm thickness is fabricated along the 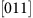 crystal axis of GaAs. (**b**) FCSEM image of the Schottky gate electrodes defining the quantum dot at the right clamping point of the mechanical resonator (scale bar, 1 μm). Application of negative bias voltage to these gates depletes the underlying two-dimensional electrons and confines a few electrons within a small spacial area of <300 × 300 nm^2^ (red circle in **b**). (**c**) Finite element method simulation of the mechanical strain associated with the fundamental flexural mode's motion, showing the maximum strain at the clamping points. (**d**) The frequency response voltage power spectrum *S*_V_ of the electromechanical transducer around the centre frequency *f*_0_=1664699.2 Hz of the fundamental flexural mode along with a Lorentzian fit (solid line). (**e**) A plot of the differential conductance of the quantum dot as a function of *V*_g_ and *V*_sd_ showing typical Coulomb diamonds as indicated by the red dashed lines. In each diamond, the number of electrons *N* in the QD are changed. In this study, we focus on the Coulomb peak enclosed by the blue square. (**f**) A Coulomb peak with the mechanical resonator under three different actuation conditions; *α*, no actuation; *β*, off-mechanical resonance actuation with *V*_d_=150 μV at *f*=*f*_0_−100 Hz; *γ*, on-mechanical resonance actuation with *V*_d_=150 μV.

The GaAs/AlGaAs modulation-doped heterostructure used in this study sustains a two-dimensional electron gas (2DEG) 90 nm below the surface and Au-Schottky gate electrodes are lithographically defined on the surface (see Methods and Supplementary Fig. 1). The two gate electrodes that are fabricated on the left clamping point of the resonator, as shown in Fig. 1a, are used to actuate and detect the mechanical motion of the resonator^14^,^25^. Figure 1d shows the measured frequency response of the fundamental flexural motion, where AC actuation voltage *V*_d_=150 μV is applied to the upper gate, while the generated piezovoltage is detected by the lower gate. From Lorentzian fitting, a resonance frequency *f*_0_=1.664699 MHz and quality factor *Q*_0_=2.4 × 10^5^ are determined. The QD is electrostatically defined using the Schottky gate electrodes on the right clamping point of the mechanical resonator as shown in Fig. 1b. The charge states in the QD can be controlled via the gate-voltage *V*_g_ and the source-drain bias *V*_sd_ applied across the QD, and are monitored via the resultant current through the QD. Figure 1e shows a grayscale plot of the differential conductance *G* as a function of *V*_g_ and *V*_sd_ showing a typical Coulomb diamond structure. In what follows, we mainly focus on the Coulomb peak around *V*_g_=−0.465 V (≡*V*_g0_) as indicated by the square in Fig. 1e.

The coupling between the QD and the resonator arises from piezoelectricity in GaAs. The elastic strain associated with the mechanical motion induces a piezoelectric field that can serve as an effective gate voltage acting on the QD. To maximize this effect, the position of the QD is designed with reference to finite element method simulation of the corresponding mechanical strain. The results (shown in Fig. 1c) suggest that the maximum strain appears at the clamping points and thus we locate the QD at this point. To confirm the mechanical coupling in this device, a Coulomb peak is measured at different mechanical actuation conditions (as shown in Fig. 1f). This figure reveals that the Coulomb peak is clearly distorted from its intrinsic shape when the resonator is actuated on mechanical resonance that is *f*=*f*_0_. This behaviour is in contrast to the case of off-mechanical resonance actuation, that is, *f*=*f*_0_−100 Hz, where the Coulomb peak shows no distortion, thus ensuring that the coupling is purely mechanical and not electric between the left and right clamping points.

In this configuration, the hybrid electromechanical system can be modelled by the Jaynes–Cummings Hamiltonian:


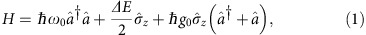


where *ħ* is the reduced Plank constant. The first term describes the mechanical resonator as a harmonic oscillator with frequency *ω*_0_=2*πf*_0_ where 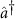 and 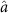 are its creation and annihilation operators. The second term describes the Pauli operator 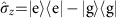 mapped onto the two-level system composed of a higher 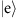 and a lower 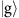 energy state in the QD with energy difference *ΔE*. The coupling between the mechanics and the QD is captured by the third term, which describes the energy shift in the two level system from the mechanical motion. This coupling can be quantified by the single phonon coupling constant *ħg*_0_=*x*_zp_(d(*ΔE*)/d*x*) where the mechanical displacement 
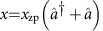
 in terms of its zero-point fluctuation *x*_zp_. Indeed, to completely define this hybrid system, equation (1) should also include terms corresponding to the conduction electrons in the source-drain leads and the tunnel coupling between the leads and the QD. These additional terms describe the situation when a finite bias voltage is applied to the source-drain leads, leading to non-equilibrium transport across the QD. Consequently, when the mechanical motion changes *ΔE* in the QD, the response of the charge transport across the QD can induce piezoelectric stress, which can act as a backaction force on to the resonator.

### Determination of the coupling constant

**Figure 2:**
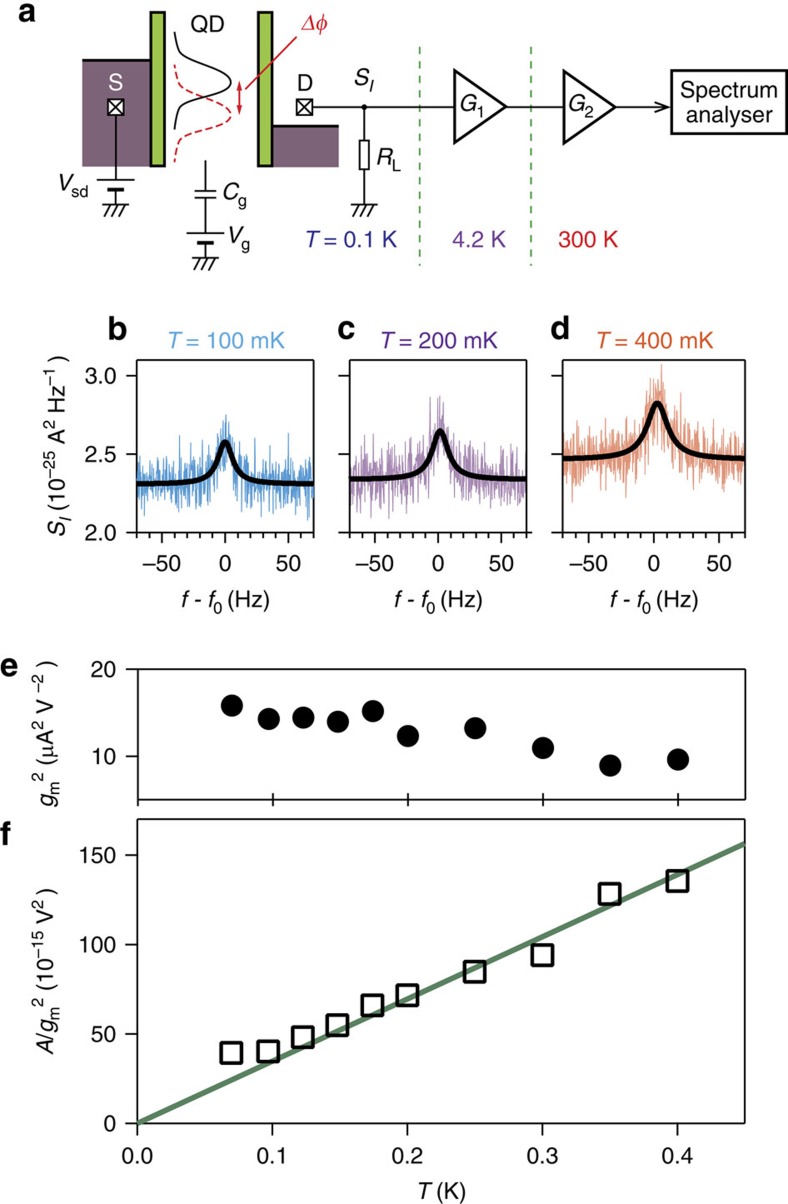
Thermal motion measured via the quantum dot current. (**a**) A schematic of the experimental setup for measuring thermal motion of the mechanical resonator. The potential fluctuation *Δϕ* induced by the piezoelectric field associated with the mechanical thermal motion results in current fluctuations, which are fed into a load resistor *R*_L_=1 kΩ and the voltage drop across the load is amplified by cryogenic (*G*_1_) and room-temperature (*G*_2_) amplifiers (see Methods). The resultant amplified signal is measured with a spectrum analyser. (**b**–**d**) The current power spectral density *S*_I_ around *f*_0_ at three different temperature. The bold solid line shows a Lorenzian fit, from which the peak area *A* corresponding to the squared amplitude of the mechanical motion is obtained. (**e**,**f**) Temperature dependence of the transconductance *g*_m_≡d*I*/d*V*_sd_ (**e**) and *A* normalized by *g*_m_ (**f**) along with a linear fit (solid line).

To quantitatively characterize the QD resonator coupling, the thermomechanical motion of the resonator was detected via the current fluctuation through the QD using the setup in Fig. 2a. Figure 2b–d show the power spectrum density at three different temperatures (100, 200 and 400 mK). The high-temperature spectrum shows a larger peak area compared with the low-temperature spectrum, reflecting the correspondingly larger thermal motion. According to the energy equipartition theorem, the squared displacement 〈*x*^2^〉 of the thermal motion is proportional to the temperature^25^. Consequently, measuring the temperature dependence of the power spectra enables the QD current to be converted into a displacement, thus yielding a responsivity. Figure 2f shows the temperature dependence of the peak area normalized by the square of the transconductance *g*_m_, which is shown in Fig. 2e. The normalized peak area linearly depends on temperature, which yields a responsivity of 1.82 × 10^6^ Vm^−1^, and this calibration enables conversion of the current noise spectra *S*_I_ into the desired displacement noise spectra. From this analysis, the minimum detectable displacement of 63 fm Hz^−0.5^ and position resolution of 170 fm are extracted. This performance is comparable to values reported using metal-based SET detectors^18^,^19^, as well as being three orders of magnitude more sensitive than a pure piezovoltage measurement from the Schottky electrode (Fig. 1d), which typically performs on the order of 10–100 pm Hz^−0.5^ as reported elsewhere^26^,^27^.

Finally, this responsivity (*η*) enables the single phonon coupling rate to be extracted from *ħg*_0_=(*Δϕ*/*ΔV*_g_)*ηΔx*_zp_, where the transfer coefficient *Δϕ*/*ΔV*_g_ defines the change in the electrostatic potential *Δϕ* in the QD induced by the effective gate voltage *ΔV*_g_ associated with the mechanical motion. In this QD, *Δϕ*/*ΔV*_g_=0.14 eVV^−1^ is determined from the Coulomb diamond detailed in Fig. 1e, which yields *g*_0_/2*π*=150 kHz. To evaluate the potency of this coupling^16^, *g*_0_ is normalized by *ω*_0_, yielding a value of ∼0.09, which is comparable to superconducting qubit-based hybrid mechanical systems where values in the range of 0.005–0.125 have been achieved^28^,^29^. Indeed, in this regime backaction from the superconducting qubit's charge states has been observed, thus suggesting the availability backaction effects in our semiconducting QD-based hybrid mechanical system.

### Backaction from the QD

The presence of backaction emerges as a perturbation of the resonance frequency *f*=*f*_0_+*Δf*_B_ and the quality factor *Q*=*Q*_0_+*ΔQ*_B_ from their intrinsic values, where a positive (negative) value of *ΔQ*_B_ indicates amplification (damping) of the mechanical motion from the backaction force. In standard models, this amplification and damping can be described in terms of a backaction force, which is characterized by a delay time 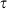. In this system, 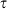 is the time for the local charges in the QD to produce a mechanical force after the mechanical motion modulates the charge distribution in the QD^2^,^30^. The resultant shift in *ω* and *Q* are approximately given by


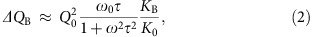



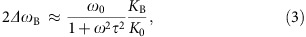


where *K*_B_ and *K*_0_ are the backaction and intrinsic force constants, respectively, with 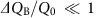 and 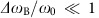. Both suppression and enhancement of *Q*-factor can then be obtained depending on the sign of *K*_B_.

**Figure 3:**
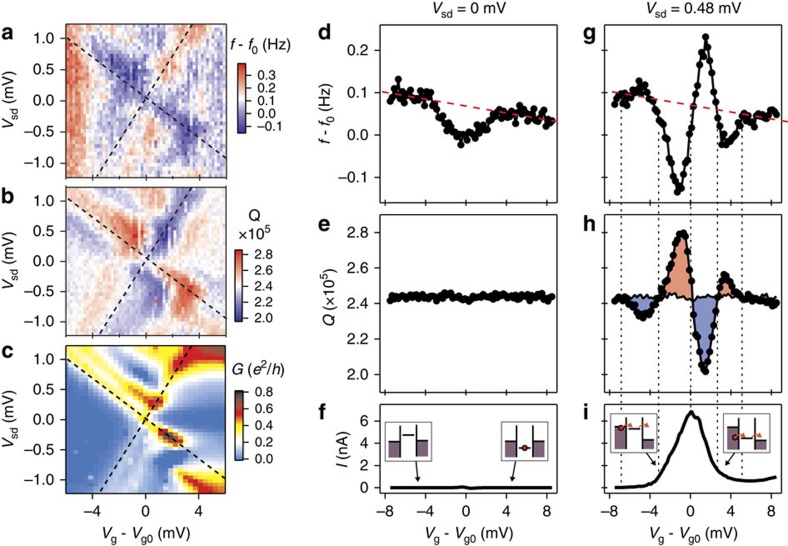
Backaction effects as a function of QD bias conditions. (**a**–**c**) Colour plots of frequency shift *f*−*f*_0_, quality factor *Q* and differential conductance *G* as a function of *V*_sd_ and *V*_g_. These plots correspond to the *V*_sd_ and *V*_g_ region as indicated by the blue square in Fig. 1e and *V*_g0_=−0.465 V is the gate voltage at which the Coulomb peak is positioned, where the dashed lines indicate the Coulomb diamond. (**d**–**i**) Comparison of the mechanical backaction effects between *V*_sd_=0 mV (**d**–**f**) and *V*_sd_=0.48 mV (**g**–**i**). In *V*_sd_=0 mV, a linear *f*-shift, as indicated by the dashed line, as well as a *f*-dip at *V*_g_=*V*_g0_ is observed, whereas no deviation is found in the corresponding *Q*-factor as *I*=0. At finite *V*_sd_, both frequency and *Q*-factor are modulated around the Coulomb peak, indicating the presence of a backaction force from the local charge state in the QD. Red (blue) area in **h** highlights the enhancement (suppression) of *Q*-factor. Schematics in (**f**,**i**) depict the corresponding energy diagram in the QD and the leads. In **f**, the relevant single electron level is above (below) the unbiased electrochemical potential when *V*_g_<*V*_g0_ (*V*_g_>*V*_g0_). In **i**, the electron energy level moves across the electrochemical potential in the source (drain) electrode when *V*_g_<*V*_g0_ (*V*_g_>*V*_g0_).

Figure 3a–c show the measured *f*-shift, *Q*, and *G* as a function of *V*_g_ and *V*_sd_, where *f* and *Q* are determined by Lorentzian fits to the frequency response at each *V*_g_ and *V*_sd_ by actuating the resonator at *V*_d_=150 μV and measuring the resultant motion via the detection circuit on the left clamping point as shown in Fig. 1a. Both *f* and *Q* deviate from their intrinsic values around the Coulomb peak, indicating the presence of backaction in this regime. Figure 3d–i show characteristic traces measured at *V*_sd_=0 and *V*_sd_=0.48 mV, highlighting the clear difference between equilibrium and non-equilibrium conditions, respectively. In Fig. 3d (*V*_sd_=0), the measured *f* shows a linear *V*_g_ dependence as indicated by the dashed line and a dip at *V*_g_=*V*_g0_, which is in contrast to the *Q*-value shown in Fig. 3e, which keeps its intrinsic value throughout the measured *V*_g_ range. The linear *f*-shift is caused by *V*_g_-induced piezoelectric strain, which linearly modifies the spring constant that in turn tunes the resonance frequency as widely observed in GaAs-based electromechanical resonators^14^,^31^. The additional dip structure at *V*_g_=*V*_g0_ is caused by a softening of the spring constant due to single-electron charge fluctuation in the QD as seen previously^22^,^23^. Specifically, the alignment of the single electron state in the QD with the electrochemical potential of the unbiased source-drain leads yields stochastic charge fluctuations whose piezoelectric backaction onto the mechanics causes a redshift in the resonance frequency.

More striking features are observed in non-equilibrium conditions with a finite *V*_sd_=0.48 mV where both *f* and *Q* nonlinearly vary around the Coulomb peak as shown in Fig. 3g–i. The sign and the magnitude of the resultant deviations depend on *V*_g_, so that the polarity and the efficiency of backaction is electrically tunable depending on the QD's energy states. As the *Q*-factor characterizes the mechanical damping properties of the resonator, the observed enhancement (suppression) of *Q* indicates an amplification (damping) of the mechanical motion driven by the single electron transport in the QD.

## Discussion

This asymmetrically oscillating perturbation feature centred at the Coulomb peak has not been observed previously in other electromechanical resonator hybrid systems. More usually, only the suppression of *Q* due to enhanced energy dissipation from single-electron fluctuations is observed in both biased metal- and carbon-nanotube-based SETs^22^,^23^,^24^,^32^. These latter observations are well explained by standard models where a single electron state in the SET is assumed to be incoherently tunnel coupled to the lead electrodes and the delay is determined by the electron tunnelling process, which is generally much faster than the resonator motion. In fact, the estimated delay time 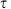
=290 ns for this semiconductor-based QD electromechanical system, obtained by substituting the observed deviations in *f* and *Q* into 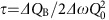, derived from equations (2) and (3), is long when compared with the typical time scales relevant to transport in QDs such as tunnelling, which is of the order of a picosecond.

Very recently, a new model was proposed assuming an additional delay time in the system, which can describe the asymmetrical oscillation of the *Q*-factor around the Coulomb peak^30^. If this time delay, *T*_12_ satisfies the condition *ω*_0_*T*_12_≈1, the timescale for mechanics and the electronic states in the QD enable the efficient manifestation of backaction effects. The possible mechanism that could lead to the additional delay process include coherent electron transfer across the QD^21^,^33^ and excitation/relaxation processes between, for instance, the first two levels in the QD^30^.

The relevance of this model to our experiment is further supported by comparing it with the backaction induced by a quantum point contact (QPC), which eliminates the energy-level structure that is suggested as being essential to the asymmetric oscillation in the *Q*-factor in the delayed backaction model. Indeed, in the QPC case *f* and *Q* shifts are also observed when finite *V*_sd_ is applied to the QPC (Supplementary Figs 2 and 3); however, their features are radically different to those observed with the QD, both qualitatively and quantitatively (see Supplemental Note 1). In particular, *f*- and *Q*-shifts show clear dependence on the power *P*=*IV*_sd_, suggesting that the observed behaviour in the QPC case originates from Joule heating (Supplementary Fig. 3d). In contrast for the QD, no *P* dependence is found as shown in Supplementary Fig. 4, thus demonstrating that the observed backaction in this case (Fig. 3g,h) stems from a mechanism other than Joule heating. Consequently, the fact that the QPC has no backaction effect (other than Joule heating) on the mechanics verifies the central role played by the excitation/relaxation process in the energy-level structure confined by the QD.

In conclusion, highly tunable backaction effects are demonstrated in a QD-mechanical resonator hybrid system integrating a gate-defined GaAs/AlGaAs QD into a piezoelectric beam resonator. Depending on the applied gate voltage, both damped and amplified mechanical motion can be activated by the single electron transport in the QD. The unique feature of this system is that the backaction polarity and the magnitude can be precisely controlled by simply tuning the operating point of the QD transport. In practice, the current-driven phonon amplification offers a key breakthrough to realizing current injection phonon lasers^12^,^13^,^34^. More fundamentally, the turnstile operation of the QD current could be harnessed in an electrically controlled single phonon emitter. Furthermore, the switchable operation from backaction cooling to parametric amplification paves the way to generating non-classical phonon states, which transfer the microscopic quantum phenomena uniquely observed in low-dimensional electron systems into macroscopic mechanical objects.

## Methods

### Sample fabrication

The hybrid QD mechanical resonator system was fabricated from a GaAs/AlGaAs modulation doped heterostructure (see Supplementary Fig. 1) sustaining a 2DEG. A single hetero junction is located 90 nm below the surface, where a 2DEG with a sheet density of 3 × 10^11^ cm^−2^ is formed. The lithographic steps employed to fabricate the hybrid device are as follows: (1) a shallow mesa containing the 2DEG was defined by photolithography and wet-etched using an H_2_O:H_2_O_2_:H_2_SO_4_ (25:1:5) solution at 10 °C for 40 s. The mesa height was 200 nm. (2) Ohmic contacts to the 2DEG were defined by means of photolithography and deposition of 200-nm-thick AuGeNi. The sample was then annealed in H_2_ ambient at 430 °C for 60 s, to alloy the AuGeNi to the 2DEG. (3) Cross-marks used as alignment markers for the electron beam (e-beam) lithography were defined by e-beam lithography and were deposited with Cr/Au (1/100 nm). (4) The fine mesa pattern near the electromechanical resonator (purple area in Fig. 1a) was defined by e-beam lithography and wet-etched as in step (1). (5) Again, using e-beam lithography, the fine Schottky electrodes at the resonators clamping point were defined and then deposited with Cr/Au (1/25 nm). (6) Photolithography was then used to define the metal lines connecting the fine Schottky gate electrodes to the bonding pads, which were deposited with 200-nm-thick Au. (7) The final photolithography step defined the doubly clamped mechanical resonator, which was wet-etched using H_2_O:H_2_O_2_:H_2_SO_4_ (25:1:5) solution at 10 °C for 5 min, to expose the Al_0.65_Ga_0.35_As sacrificial layer. The resonator was then released from the substrate by selectively etching the sacrificial layer using hydrofluoric acid (HF) solution (10 wt%) for 4 min at room temperature. After rinsing the sample in water, acetone and ethanol, the sample was gently dried in air.

### Measurement setup

All measurements were carried out inside a high-vacuum chamber (<10^−5^ Pa) on a dilution refrigerator with a base temperature of 80 mK. The thermal motion measurement of the resonator through the QD current (data shown in Fig. 2) was carried out using the setup shown in Fig. 2a, where a bias voltage *V*_sd_ is applied to the source contact and the drain is shunted to the ground by a load resistor *R*_L_=1 kΩ. A voltage drop across the load is amplified by a home-made HEMT amplifier cooled at 4 K and is followed by a commercially available amplifier (NF SA-220F5) at room temperature. The total voltage power gain factor *F* of this two-stage setup was *F*=7.32 × 10^6^ in units of V^2^ V^−2^. The amplified voltage signal is fed to a digital spectrum analyser and is converted to a frequency-domain spectrum. The measured power spectrum *S*_out_ of the amplified voltage signal is related to the power spectrum of the QD current *S*_I_ via 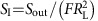. The mechanical motion measurements shown in Figs 1d and 3d–i were carried out by measuring the piezovoltage generated on the electrodes at the left-clamping point (Fig. 1a) using a two-stage amplification setup similar to that detailed above.

### Thermal motion analysis

The power spectrum density of the QD current *S*_I_ is related to the displacement spectra *S*_*x*_ of the resonator via 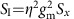. Here, *η* is the responsivity, which is the transfer coefficient from the displacement of the mechanical resonator to the piezoelectric gate voltage that appears on the QD, and *g*_m_≡d*I*/d*V*_g_ is the transconductance, that is, the transfer coefficient from the piezoelectric gate voltage to the QD current. According to the energy equipartition theorem^19^, the squared displacement 〈*x*^2^〉 of the thermal motion is proportional to *T* as *K*_s_〈*x*^2^〉=*k*_B_*T* with two known parameters; *K*_s_ being the spring constant and *k*_B_ being the Boltzmann constant. In this resonator, *K*_s_=128.4 Nm^−1^ was deduced from *K*_s_=*M*_eff_(2*πf*_0_)^2^ with *M*_eff_=1.174 × 10^−12^ kg being the effective mass of the resonator. Here *M*_eff_=0.73*M* and *M* is the total mass of the beam; the coefficient 0.73 normalizes the fundamental flexural mode of the doubly clamped beam resonator to a harmonic oscillator^35^. Care was exercised in calibrating the net responsivity *η* from the *T* dependence of the thermal motion spectra (Fig. 2b–d), because thermal broadening of the Coulomb peak also reduces the transconductance *g*_m_ (Fig. 2e). To correct for this reduction, *g*_m_ was measured as a function of temperature with a small AC voltage modulation of 10 μV at 13 Hz superposed to the DC gate voltage *V*_g_, which yielded a modulated current that was detected in a lock-in amplifier. Next, the reduced *g*_m_ was accounted for by normalizing the peak area *A* by 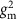. Figure 2f shows that 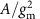 depends linearly on *T*, reflecting the linear *T* dependence of the thermal motion. The slope of the linear fit, combined with the equipartition theorem, namely 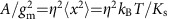, allows the responsivity *η*=1.82 × 10^6^ Vm^−1^ to be deduced. From this, the current power spectrum *S*_I_ in the thermal motion spectra (Fig. 2b–d) can be converted to the corresponding displacement *S*_*x*_ using 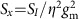.

The lowest noise floor observed at *T*=80 mK corresponds to 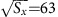 fm Hz^−0.5^, which is the minimum detectable displacement sensitivity in the present setup. The position resolution *δx* is also estimated to be *δx*=170 fm, using (*δx*)^2^=*S*_*x*_ × *Δf* with *Δf*=*f*_0_/*Q* being the bandwidth of the resonator. This optimum position resolution is ∼70 times the zero-point fluctuation 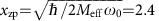 fm for this resonator.

## Additional information

**How to cite this article:** Okazaki, Y. *et al*. Gate-controlled electromechanical backaction induced by a quantum dot. *Nat. Commun.* 7:11132 doi: 10.1038/ncomms11132 (2016).

## Supplementary Material

Supplementary Information Supplementary Figures 1-4 and Supplementary Note 1
