# Supplementary material for: Gate-controlled electromechanical backaction induced by a quantum dot
Source: Nat Commun. 2016 Apr 11;7:11132. doi: 10.1038/ncomms11132 (PMC4831016; doi:10.1038/ncomms11132)
Supplement: Supplementary Information — Supplementary Figures 1-4 and Supplementary Note 1 [file ncomms11132-s1.pdf]

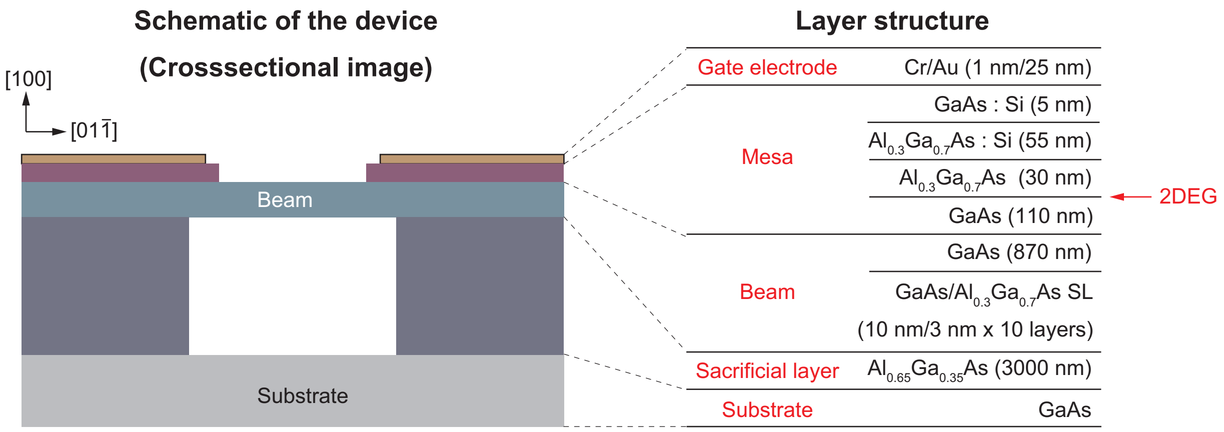

SUPPLEMENTARY FIGURE 1. A schematic of a cross-section of the GaAs/AlGaAs modulation doped heterostructure used in this study.

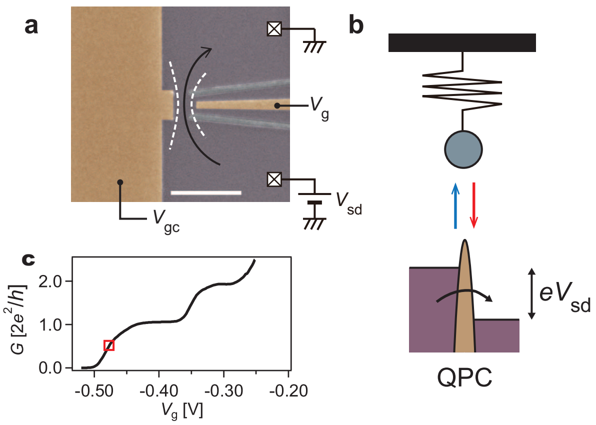

SUPPLEMENTARY FIGURE 2. The quantum point contact. (**a**) The gate bias conditions used for defining a quasi-one dimensional transport channel, namely a QPC (scale bar, 1 μm). (**b**) A schematic of the electromechanical resonator and QPC hybrid setup, in which the mechanical motion influences the QPC's potential barrier through which electrons pass. (**c**) A plot of the differential conductance G as a function of V_g measured at $V_{sd} = 1$ mV.

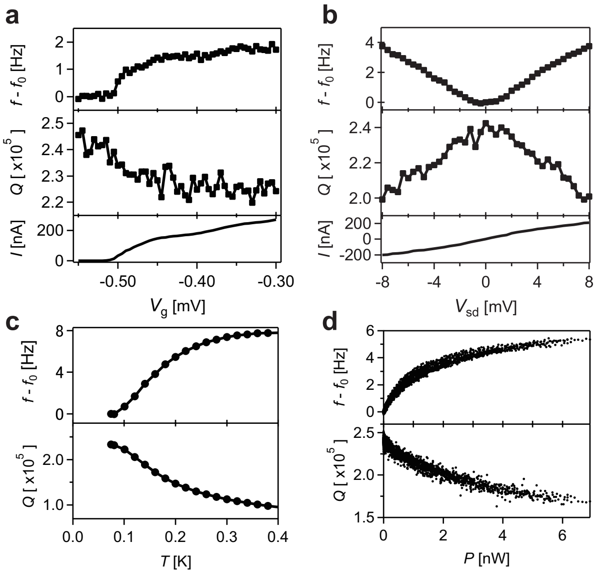

SUPPLEMENTARY FIGURE 3. Backaction from the QPC. (**a**) The V_g-dependence of the frequency shift $f - f_0$, Q-factor and dc current I measured at $V_\mathrm{sd} = 2.4$ mV. (**b**) The V_sd-dependence of $f - f_0$, Q and I measured at $V_\mathrm{g} = -0.48$ V (square in Supplementary Fig. 2c). (**c**) The temperature dependence of $f - f_0$ and Q show a positive frequency shift and suppression of Q with increasing T. (**d**) Joule power $P = V_\mathrm{sd} I$ dependence of f and Q, all data points are measured at 51 different V_g values for which the QPC conductance increases from 0 to 2.5 $2\mathrm{e}^2/\mathrm{h}$.

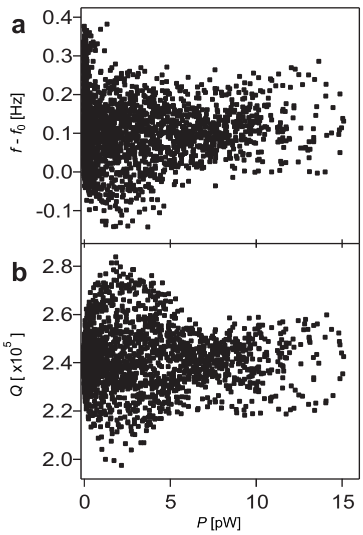

SUPPLEMENTARY FIGURE 4. Absence of heating in the QD backaction. Joule power $P = V_\mathrm{sd} I$ dependence of (**a**) f and (**b**) Q in the QD case confirms the absence of backaction from Joule heating. All data points are extracted from Figs. 3a and b.

SUPPLEMENTARY NOTE 1: BACK-ACTION FROM THE QPC

In order to confirm the impact of the local charge states in the QD on the observed enhancement/suppression of Q, we compare it with the case using a quantum point contact (QPC). By grounding the two side gates, local charges confined in the gate-enclosed area are eliminated, instead, a quasi-one-dimensional tunneling barrier is formed between the two remaining gates (see Supplementary Fig. 2a), known as a QPC. The formation of the QPC can be confirmed via the quantized conductance, which emerges as equally-separated current steps as a function of V_{g} as shown in Supplementary Fig. 2c.

In the case of the QPC, the mechanical motion can modulate the tunneling barrier and the electrons perturbed by the mechanical motion are rapidly transported to ground without dwelling in the proximity of the mechanical resonator thus imposing no backaction onto the mechanics (see Supplementary Fig. 2b). This situation is radically different to the QD case where the transiting electrons dwell in the QD and thus the corresponding localised charges can induce a backaction on the resonator's motion. To highlight this difference, Supplementary Fig. 3a shows the measured f and Q as a function of V_{g} at $V_{\mathrm{sd}} = 2.4$ mV, which reveals that f (Q) blue shifts (is suppressed) as the QPC current I increases. Similar deviations in f and Q are also observed at different V_{sd} as shown in Supplementary Fig. 3b. As detailed above, this observation is different from that of the QD both qualitatively and quantitatively. First, no enhancement in Q is observed over a wide V_{g}- and V_{sd}-range ($-0.55 < V_{\mathrm{g}} < -0.3$ V and $|V_{\mathrm{sd}}| < 8$ mV). Second, the observed f-shift (typically $f - f_0 > 1$ Hz) is almost an order of magnitude larger than that observed in the QD case (typically $f - f_0 \approx \pm 0.1$ Hz as shown in Figs. 3a, d, and g). These two differences suggest that the backation from the QPC arises from a different origin. Supplementary Fig. 3c shows the temperature dependence of f and Q revealing a positive f-shift and suppression of Q with increasing system temperature. This observed temperature-dependence quantitatively agrees with the aforementioned behavior in f and Q, indicating that the QPC induced backaction can be attributed to current-induced Joule heating, which is capable of changing the mechanical properties of the resonator. This assertion is further strengthened when the observed f-shift and the change in Q are plotted as a function of the heating power $P = V_{\mathrm{sd}} I$ as shown in Supplementary Fig. 3d. Here mechanical resonance traces are measured at 51 different V_{g} values for which the QPC conductance varies from 0 to 2.5 $2e^2/h$ at each V_{sd} namely P. Although f and Q are measured with different QPC conductance that is as a function of V_{g}, all data points shown in this plot clearly only depend on P.

To compare this result with the case using the QD, similar plots for f and Q as a function of P are shown in Supplementary Fig. 4, where f and Q appear to be random and no clear dependence on P can be observed. From this comparison, we conclude that the QPC has no back-action effects akin to that observed for the QD but instead hosts Joule heating induced-deviation of f and Q whereas the backaction from the QD stems from a mechanism other than Joule heating.
